# H5 subtype avian influenza virus induces Golgi apparatus stress response via TFE3 pathway to promote virus replication

**DOI:** 10.1371/journal.ppat.1012748

**Published:** 2024-12-09

**Authors:** Yuncong Yin, Xianjin Kan, Xinyu Miao, Yingjie Sun, Sujuan Chen, Tao Qin, Chan Ding, Daxin Peng, Xiufan Liu

**Affiliations:** 1 College of Veterinary Medicine, Yangzhou University, Yangzhou, Jiangsu, PR China; 2 Jiangsu Co-Innovation Center for the Prevention and Control of Important Animal Infectious Disease and Zoonoses, Yangzhou, Jiangsu, PR China; 3 Jiangsu Research Centre of Engineering and Technology for Prevention and Control of Poultry Disease, Yangzhou, Jiangsu, PR China; 4 Department of Avian Infectious Diseases, Shanghai Veterinary Research Institute, Chinese Academy of Agricultural Science, Shanghai, PR China; 5 Shanghai Jiaotong University School of Agriculture and Biology, Shanghai, PR China; 6 The International Joint Laboratory for Cooperation in Agriculture and Agricultural Product Safety, Ministry of Education, Yangzhou University, PR China; The Ohio State University, UNITED STATES OF AMERICA

## Abstract

During infection, avian influenza virus (AIV) triggers endoplasmic reticulum (ER) stress, a well-established phenomenon in previous research. The Golgi apparatus, situated downstream of the ER and crucial for protein trafficking, may be impacted by AIV infection. However, it remains unclear whether this induces Golgi apparatus stress (GAS) and its implications for AIV replication. We investigated the morphological changes in the Golgi apparatus and identified GAS response pathways following infection with the H5 subtype AIV strain A/Mallard/Huadong/S/2005. The results showed that AIV infection induced significant swelling and fragmentation of the Golgi apparatus in A549 cells, indicating the presence of GAS. Among the analyzed GAS response pathways, TFE3 was significantly activated during AIV infection, while HSP47 was activated early in the infection process, and CREB3-ARF4 remained inactive. The blockade of the TFE3 pathway effectively inhibited AIV replication in A549 cells and attenuated AIV virulence in mice. Additionally, activation of the TFE3 pathway promoted endosome acidification and upregulated transcription levels of glycosylation enzymes, facilitating AIV replication. These findings highlight the crucial role of the TFE3 pathway in mediating GAS response during AIV infection, shedding light on its significance in viral replication.

## Author summary

Influenza virus infection is known to trigger the endoplasmic reticulum stress response, a critical cellular reaction. The Golgi apparatus, positioned downstream of the endoplasmic reticulum, plays a vital role in protein modification and trafficking. Our study demonstrated that avian influenza virus (AIV) infection indeed induced GAS, as evidenced by the swelling and fragmentation of the Golgi apparatus observed in A549 cells. Moreover, the TFE3 pathway was significantly activated in response to GAS during AIV infection, which facilitates viral replication by enhancing endosome acidification and promoting the transcription of glycosylation enzymes. Inhibition of the TFE3 pathway significantly impeded AIV replication and decreased viral pathogenicity, suggesting its potential as a broad-spectrum target for antiviral drug development. These findings provide the first evidence linking AIV replication with GAS response, thereby advancing our understanding of AIV replication mechanisms.

## Introduction

The Golgi apparatus, an essential organelle ubiquitous in eukaryotic cells and integral to the endomembrane system within the cytoplasm, plays a pivotal role in protein trafficking [1]. Serving as a crucial hub at the nexus of secretory, lysosomal, and endocytic pathways [2–6], the Golgi apparatus holds particular significance in processing secretory proteins through glycosylation [7,8].

Comparable to the endoplasmic reticulum (ER) stress response or the unfolded protein response, which aim to restore ER homeostasis and protein folding capacity, Golgi apparatus stress (GAS) response ensues when substantial quantities of unmodified proteins accumulate within the Golgi apparatus [9]. GAS response manifests through three distinct signaling pathways: TFE3, HSP47, and CREB3-ARF4 [10]. Typically, TFE3 remains sequestered in the cytoplasm by 14-3-3 proteins in a highly phosphorylated state. Activation of the TFE3 pathway occurs during compromised Golgi function due to the accumulation of secreted glycoproteins and an excess of improperly glycosylated proteins [10–12]. Upon dephosphorylation, TFE3 translocates into the nucleus, binding to GAS response-enhancing elements and subsequently initiating the transcription of various post-translational modification enzymes, including glycosyltransferases [11]. The HSP47 pathway assumes an anti-apoptotic role during GAS response [13], initially identified through the inhibition of mucin-type O-glycosylation, a pivotal post-translational protein modification. Conversely, the CREB3-ARF4 pathway instigates apoptosis by transcribing ARF4 during GAS response [14], specifically triggered by brefeldin A (BFA), a natural compound renowned for its antiviral, antibiotic, and antifungal properties, disrupting ER-to-Golgi transport, protein secretion, and Golgi complex organization.

The H5 subtype of highly pathogenic avian influenza virus (AIV) poses significant threats to both avian populations and human health, precipitating substantial losses in the poultry industry and endangering public health on a global scale [15–18]. Notably, hemagglutinin (HA) and neuraminidase (NA) represent the principal surface glycoproteins of AIVs. These glycoproteins undergo translation and subsequent modification within the ER before progressing to the Golgi apparatus during infection [19,20]. While influenza virus infection has been shown to induce ER stress [20–23], its potential to induce GAS remains underexplored. Given the intricate interplay between viral infections and GAS, this study aims to elucidate the impact of AIV infection on the Golgi apparatus and explore the potential involvement of GAS in viral replication.

## Results

### AIV infection induces Golgi apparatus collapse at late stage of infection regardless of ER stress

**Fig 1:**
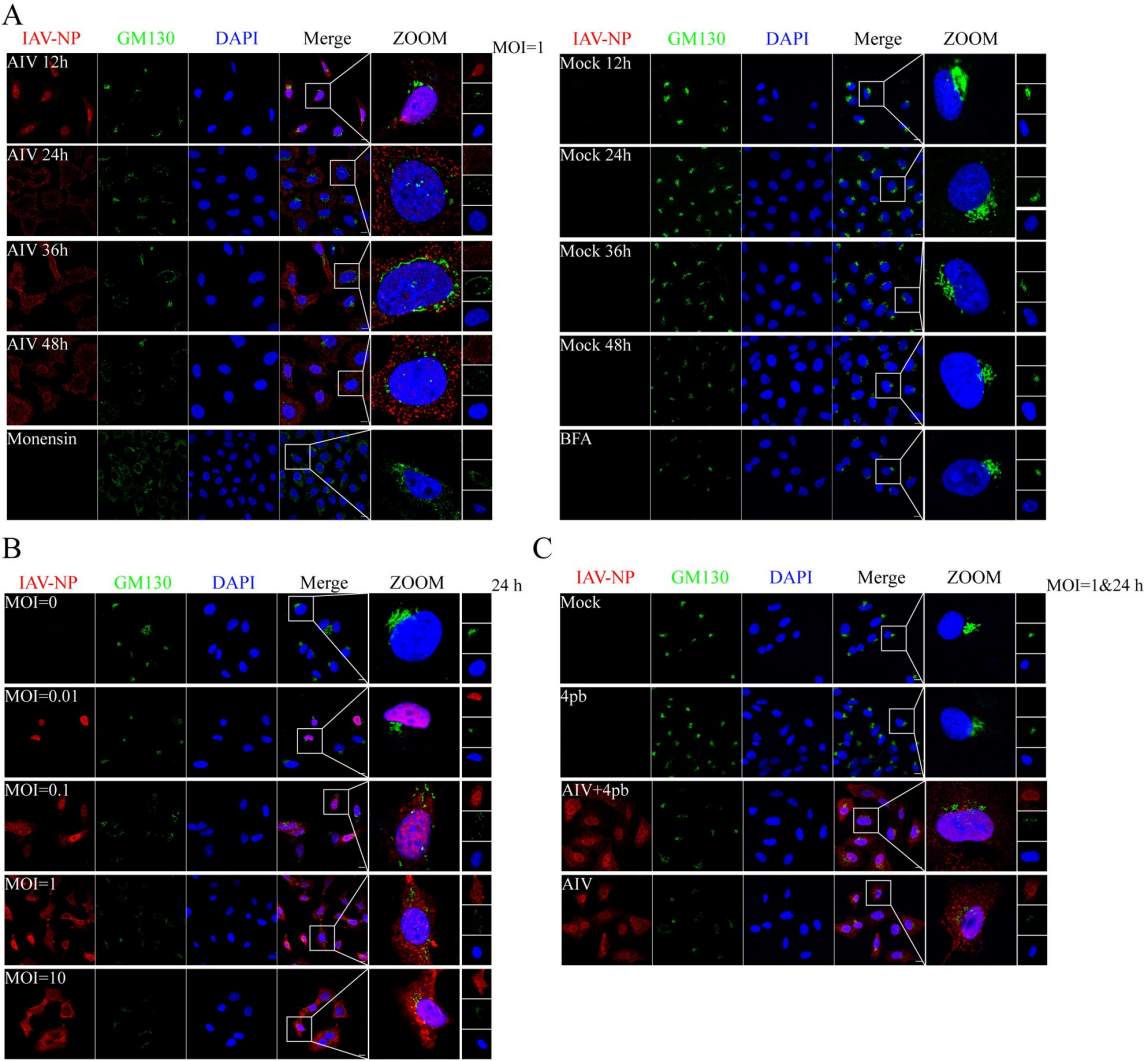
Golgi apparatus morphology after AIV infection. (A) Collapse of the Golgi apparatus induced by AIV infection at different time points. A549 cells were infected with AIV at 1 MOI, fixed on slides at 12, 24, 36, and 48 h post-infection, and incubated with antibodies against IAV-NP, GM130, and DAPI. Monensin (10 μM) treatment and BFA (10 μM) treatment served as different positive controls. (B) Collapse of the Golgi apparatus induced by AIV infection at different infection doses. A549 cells were infected with AIV at 0, 0.01, 0.1, 1, and 10 MOI, fixed on slides at 24 h post-infection, and incubated with antibodies against IAV-NP, GM130, and subjected to DAPI staining. (C) ER stress does not influence the collapse of the Golgi apparatus following AIV infection. A549 cells were infected with AIV at 1 MOI and supplemented with 4-phenylbutyric acid (5 μM), fixed at 36 h post-infection on slides, and incubated with antibodies against IAV-NP, GM130, and subjected to DAPI staining. The cells were visualized using a Zeiss confocal fluorescence microscope LSM880. Scale bar = 10 μm.

The morphology of the Golgi apparatus in A549 cells infected with A/Mallard/Huadong/S/2005 AIV was assessed using GM130 staining at 12-, 24-, 36-, and 48-hours post-infection. Notably, the Golgi apparatus exhibited a diffused state at 24-, 36-, and 48-hours post-AIV infection, with the entire fragmented Golgi closely juxtaposed to the nucleus. This morphology closely resembled the typical characteristics of stressed Golgi apparatus observed after Monensin treatment, as depicted in Fig 1A, although distinct from that of BFA-treated cells. In contrast, Golgi apparatus in mock-infected cells exhibited an aggregated state distributed exterior to the nucleus (Fig 1A). Quantitative analysis of Golgi fragmentation revealed that Golgi began collapsing at 24 hours post-AIV infection (S1A Fig). Additionally, the Golgi complex progressively collapsed into a diffused state at 24 hours post-infection with escalating AIV doses (Fig 1B).

Given the Golgi apparatus’ downstream relationship with the ER, it was hypothesized that AIV-induced ER stress might precipitate GAS. To test this hypothesis, 4-phenylbutyric acid was employed to inhibit ER stress, and Golgi morphology was observed following AIV infection. Phosphorylation of IRE1α served as confirmation of ER stress inhibition. 4-phenylbutyric acid could partially inhibit AIV induced ER stress (S2 Fig). Surprisingly, results indicated that AIV infection led to similar fragmentation of the Golgi apparatus in A549 cells with or without 4-phenylbutyric acid treatment. Notably, treatment with 4-phenylbutyric acid had no discernible effects on the morphology of the Golgi apparatus (Fig 1C), suggesting that GAS may occur independently of ER stress.

Furthermore, Golgi apparatus morphology was assessed following infection with other subtype influenza viruses, namely, A/California/04/09 (CA04, H1N1), A/Puerto Rico/8/1934 (PR8, H1N1), A/chicken/Taixing/10/2010 (TX, H9N2), and A/Chicken/Guangdong/04/2017 (GD, H7N9). Intriguingly, all these strains induced a diffused Golgi apparatus morphology at 36 hours post-infection (S3 Fig), suggesting that multiple subtypes of influenza viruses can elicit GAS.

### AIV infection activates GAS response mainly through the TFE3 pathway

**Fig 2:**
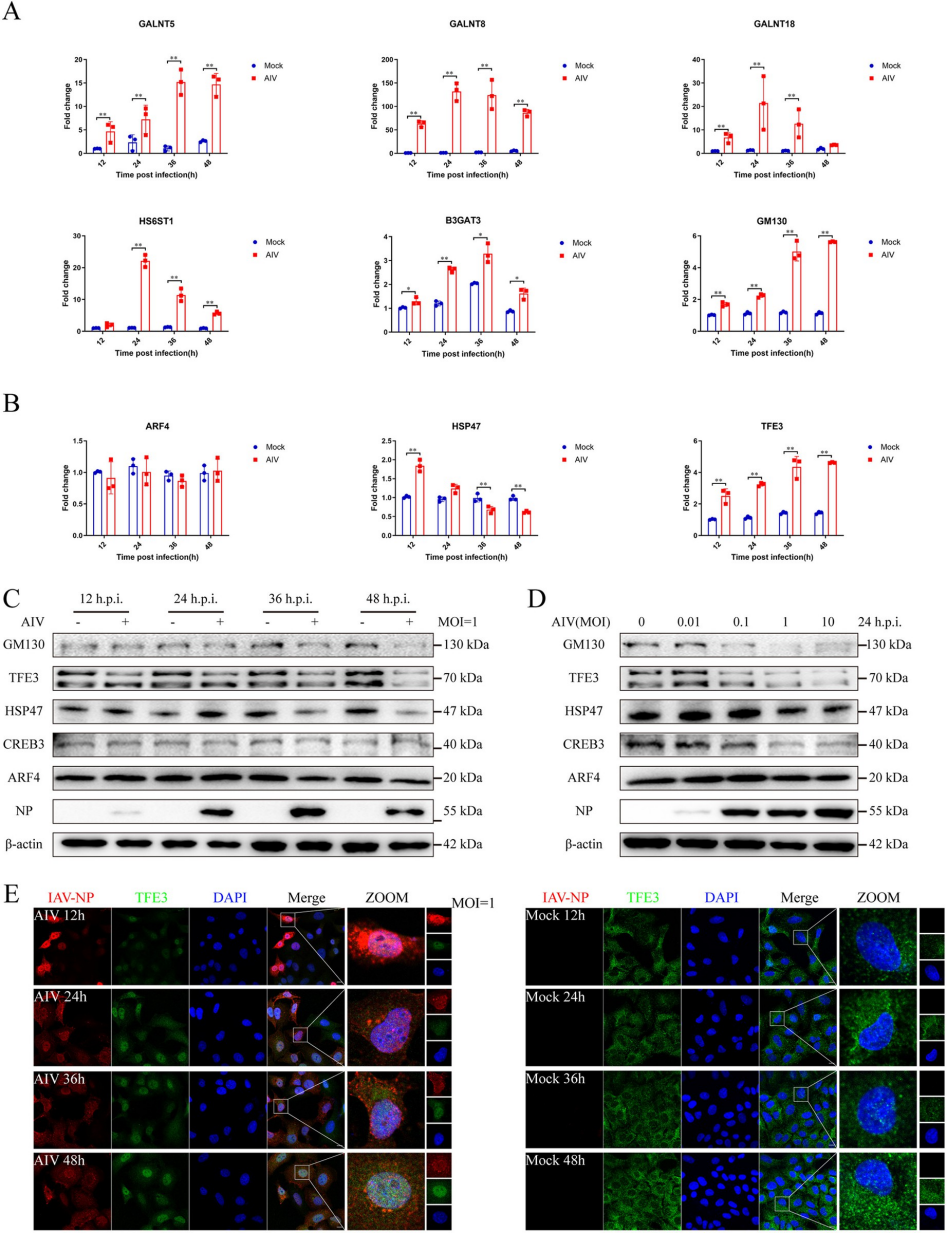
Activation of Golgi apparatus stress (GAS) response pathways following AIV infection. (A) mRNA levels of GAS-related proteins in response to AIV infection. A549 cells were infected with AIV, and total RNA was extracted at 12, 24, 36, and 48 h post-infection for qRT-PCR analysis. (B) mRNA levels of members of the GAS response pathway. A549 cells were infected with AIV at 1 MOI, and total RNA was extracted at 12, 24, 36, and 48 h post-infection for qRT-PCR analysis. (C) Expression of proteins related to GAS response pathways following AIV infection. A549 cells were infected with AIV at 1 MOI, cell lysates were prepared at 12, 24, 36, and 48 hpi, and incubated with antibodies against IAV-NP, GM130, CREB3, TFE3, HSP47, and ARF4 at 4°C overnight. β-actin served as an internal standard. Bands were visualized using a chemiluminescence imaging analysis system after incubation with peroxidase-conjugated secondary antibodies. (D) Impact of infection doses on the expression of proteins related to GAS response pathways. A549 cells were infected with AIV at 0, 0.01, 0.1, 1, and 10 MOI for 24 h, and cell lysates were prepared and incubated with antibodies against IAV-NP, GM130, CREB3, TFE3, HSP47, and ARF4 at 4°C overnight. β-actin served as an internal standard. Bands were visualized using a chemiluminescence imaging analysis system after incubation with peroxidase-conjugated secondary antibodies. (E) AIV infection-induced nuclear translocation of TFE3. A549 cells were infected with AIV at 1 MOI, fixed at 12, 24, 36, and 48 h post-infection on slides, and incubated with antibodies against IAV-NP and TFE3, followed by DAPI staining. Cells were visualized using a Zeiss confocal fluorescence microscope LSM880 (IAV-NP, red; TFE3, green; DAPI, blue). Scale bar = 10 μm. Error bars represent SD of the means from three independent experiments (*p<0.05, **p<0.01).

To explore the impact of AIV infection on GAS response, we examined the mRNA levels of key enzymes involved in protein modification within the Golgi apparatus. Specifically, we assessed the expression of polypeptide N-acetylgalactosaminyltransferase 5 (*GALNT5*), polypeptide N-acetylgalactosaminyltransferase 8 (*GALNT8*), polypeptide N-acetylgalactosaminyltransferase 18 (*GALNT18*), heparan-sulfate 6-O-sulfotransferase 1 (H*S6ST1*), and galactosylgalactosylxylosylprotein3-beta-glucuronosyltransferase 3 (*B3GAT3*). Following AIV infection, mRNA levels of *GALNT5*, *GALNT8*, *GALNT18*, *HS6ST1* and *B3GAT3* significantly increased compared to mock-infected cells (Fig 2A). Additionally, the mRNA expression of *GM130*, a structural protein of the Golgi apparatus, exhibited a significant increase at 12-, 24-, 36-, and 48-hours post AIV infection (Fig 2A). However, GM130 protein levels displayed a decreasing trend with prolonged infection duration and increasing AIV doses (Figs 2C and 2D, and S4A and S4B).

Subsequently, we investigated the activation status of the three principal GAS response pathways: CREB3-ARF4, HSP47, and TFE3. The activation of ARF4 expression served as a crucial indicator for the CREB3-ARF4 pathway. Analysis revealed that *ARF4* mRNA expression showed no significant difference compared to mock-infected cells across the infection time course (Fig 2B). Similarly, ARF4 protein levels remained consistent across different infection durations and AIV doses. Although there was a notable decrease in CREB3 protein at 24-hours post-AIV infection at both 1 MOI and 10 MOI (Figs 2D and S4B). The results still indicated that the CREB3-ARF4 pathway was not activated during AIV infection.

Moreover, *HSP47* mRNA and protein levels were significantly reduced at 36 and 48 hours post-AIV infection (Figs 2B and 2C, and S4A). HSP47 protein level increased significantly at 0.01 MOI and 0.1 MOI but decreased at 10 MOI at 24 hours post-AIV infection, suggesting a slight activation of the HSP47 pathway at the early stages of AIV infection, followed by suppression at later stages (Figs 2D and S4B).

Importantly, *TFE3* mRNA levels significantly increased at 12-, 24-, 36-, and 48- hours post-AIV infection, with a subsequent decrease in TFE3 protein levels observed at 36- and 48-hours post-infection compared to mock-infected cells (Fig 2B and 2C). Notably, dephosphorylation and nuclear translocation of TFE3 are indicative of TFE3 pathway activation. While TFE3 was primarily cytoplasmic in mock-infected cells (Fig 2E), nuclear localization and increased staining was evident at all time points post-AIV infection, suggesting considerable nuclear translocation of TFE3 in the AIV infection group (Fig 2E).

Furthermore, TFE3 nuclear translocation was assessed following infection with other subtype influenza viruses (A/California/04/09 (CA04, H1N1), A/Puerto Rico/8/1934 (PR8, H1N1), A/chicken/Taixing/10/2010 (TX, H9N2), and A/Chicken/Guangdong/04/2017 (GD, H7N9)). All these strains induced TFE3 nuclear translocation at 36 hours post-infection (S5 Fig). Collectively, these findings underscore the activation of the TFE3 pathway during AIV infection.

### Activation of the TFE3 pathway facilitates AIV replication

**Fig 3:**
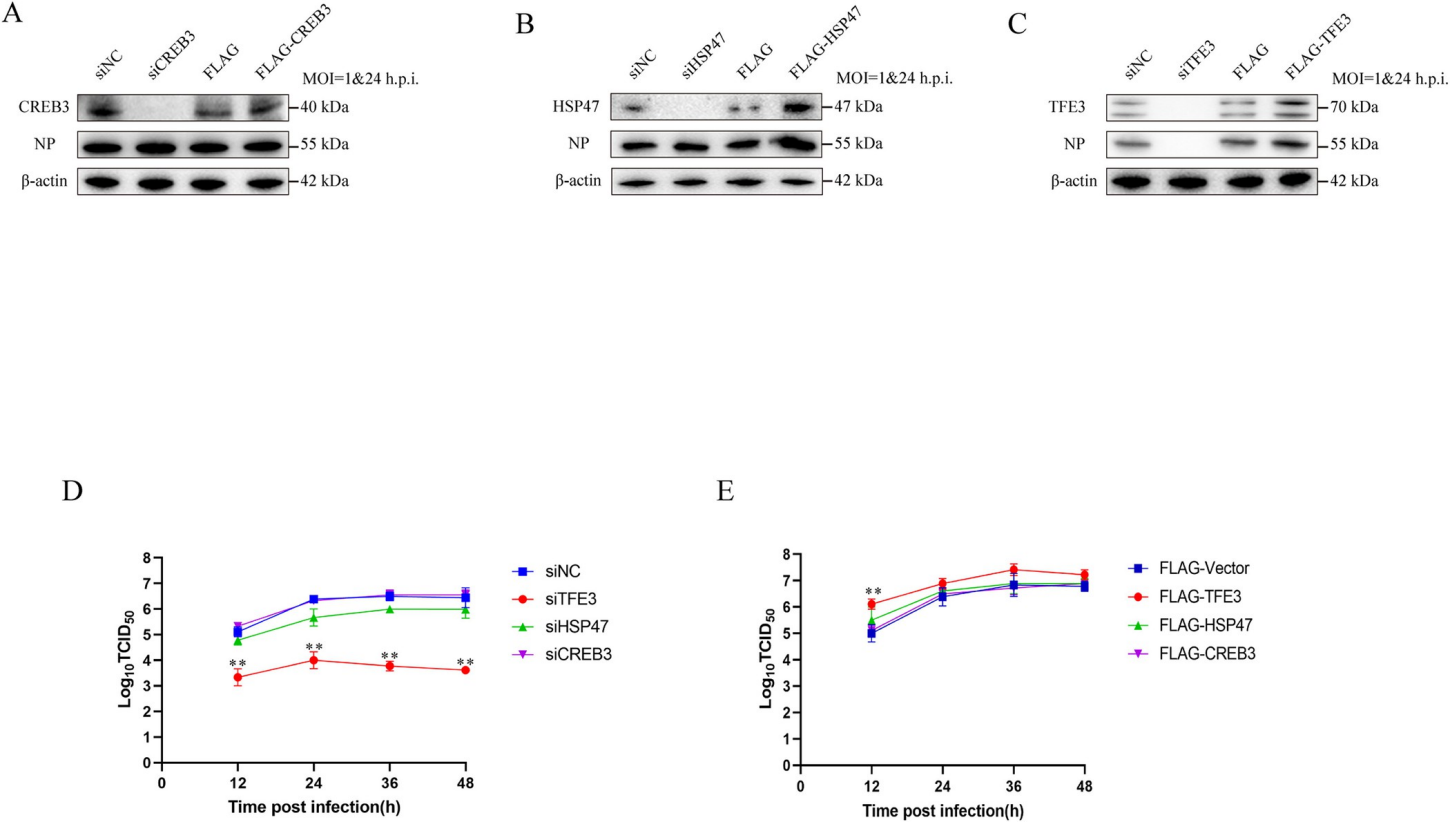
Effect of Golgi apparatus stress (GAS) response on AIV replication. Effect of knockdown or overexpression of GAS-related genes on AIV replication in A549 cells. Prior to AIV infection, cells were transfected with siCREB3 or FLAG-CREB3 (A), siHSP47 or FLAG-HSP47 (B) and siTFE3 (A) or FLAG-TFE3 (C). Cell lysates were then incubated with antibodies against ARF4, HSP47, and TFE3 to confirm RNA interference, followed by incubation with antibodies against NP. β-actin served as an internal standard. Cell supernatants with siCREB3, siHSP47, and siTFE3 (D) or FLAG-CREB3, FLAG-HSP47, or FLAG-TFE3 (E) were subjected to TCID_50_ determination at 12, 24, 36, and 48 h post-infection for viral growth curve analysis. Error bars represent SD of the mean from three independent experiments (*p<0.05, **p<0.01).

To elucidate the involvement of the GAS response in AIV replication, siRNAs targeting the CREB3-ARF4, HSP47, and TFE3 pathways were employed. Silencing of CREB3 exhibited no discernible impact on AIV replication, as evidenced by NP expression and viral growth curves in siCREB3-treated cells, which mirrored those observed in siNC-treated cells (Fig 3A and 3D). HSP47 knockdown resulted in a non-significant reduction in NP expression and viral titers (Fig 3B and 3D). Intriguingly, TFE3 knockdown led to a complete absence of detectable NP expression, accompanied by a significant decrease in viral titers compared to siNC-treated cells (Fig 3C and 3D). Moreover, overexpression of CREB3 failed to influence NP expression nor viral titers following AIV infection, with outcomes comparable to FLAG-vector transfected cells (Fig 3A and 3E). While HSP47 overexpression exhibited an increasing trend in NP expression and viral titers post-AIV infection, the differences did not reach statistical significance (Fig 3B and 3E). Importantly, TFE3 overexpression significantly augmented NP expression and viral titers following AIV infection (Fig 3C and 3E), underscoring the facilitative role of the TFE3 pathway in AIV replication.

Given that TFE3 knockdown resulted in the absence of detectable NP expression, we extended our analysis to include the expression of HA and M1 of AIV. Remarkably, TFE3 knockdown corresponded with decreased expression of both HA and M1 of AIV (S6A Fig). Additionally, we investigated the replication dynamics of other viruses, including NDV, VSV, and HSV, post-TFE3 knockdown. Consistently, TFE3 knockdown led to a significant decrease in NP expression of NDV, VSV, and HSV (S6B Fig), further emphasizing the broad impact of TFE3 on viral replication.

### Blocking the TFE3 pathway attenuates AIV replication and virulence in mice

**Fig 4:**
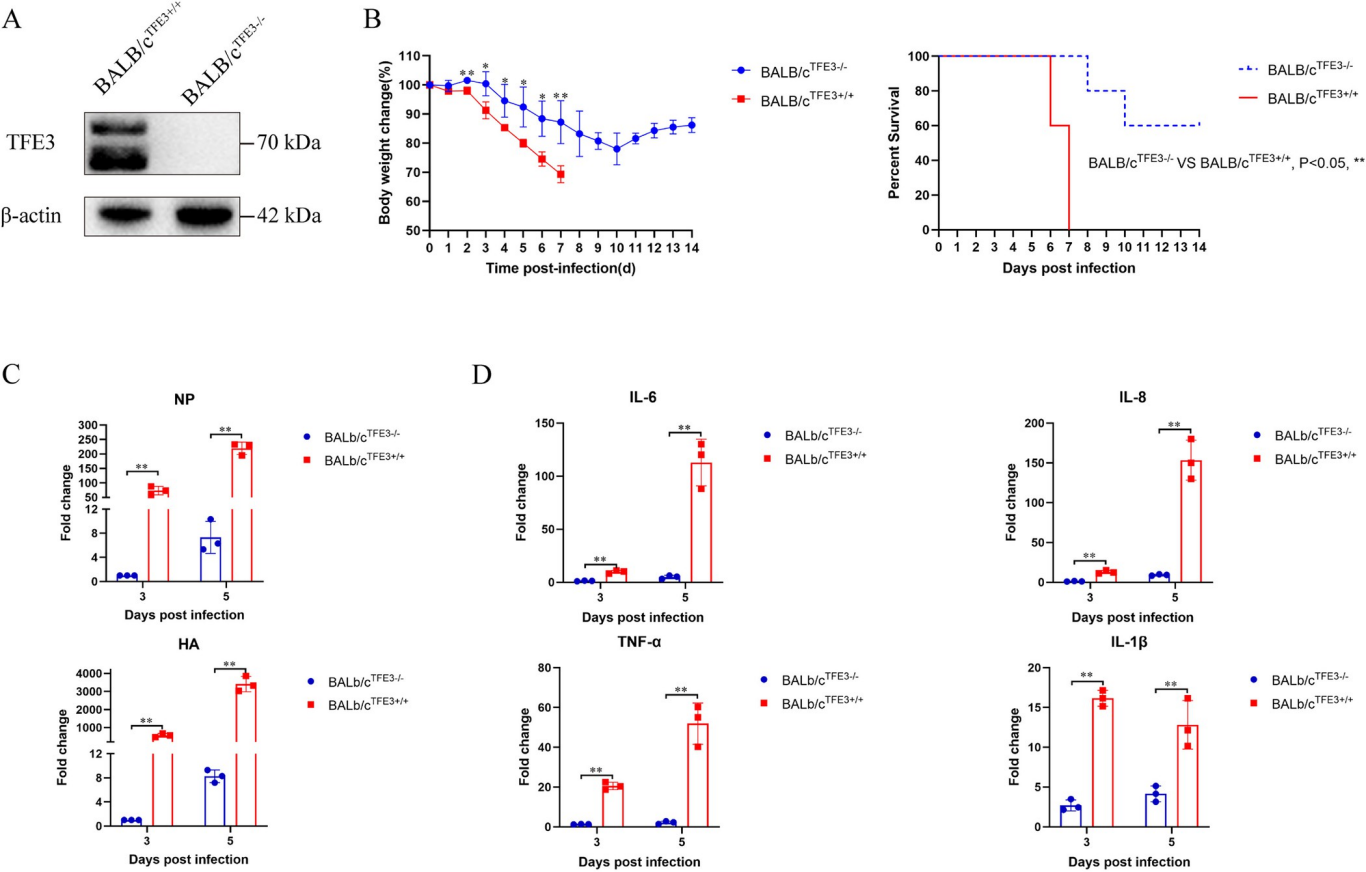
Effect of mouse *TFE3* knockout on AIV replication and virulence. (A) Confirmation of TFE3 knockout in mice using western blot analysis. Lungs from 6-week-old BALB/c^TFE3+/+^ and BALB/c^TFE3-/-^ mice were collected, and their proteins were extracted. Western blotting was performed on protein samples using an antibody against TFE3, with β-actin serving as an internal standard. Bands were visualized using a chemiluminescence imaging analysis system after incubation with peroxidase-conjugated secondary antibodies. (B) Six-week-old BALB/c^TFE3+/+^ and BALB/c^TFE3-/-^ mice were intranasally infected with 10^6^ EID_50_ of virus in 50 μL PBS. Mice were monitored daily for weight loss and signs of disease over a 14-day period. The mean body weight change (%±SD) and death rate were presented. A similar experimental set of mice was prepared, and lungs were collected on day 5 post-infection. mRNA levels of virus gene (C) and inflammatory cytokines (D) were detected using qRT-PCR. Error bars represent SD of the means of data from three independent mice (*p< 0.05, **p<0.01).

To assess the impact of TFE3 on AIV replication and virulence in vivo, TFE3 knockout BALB/c^TFE3-/-^ mice were obtained from GemPharmatech Corporation and confirmed via western blotting (Fig 4A). Upon infection with a dose of 10^6^ EID_50_, all BALB/c^TFE3+/+^ mice exhibited a considerable decrease in body weight and succumbed to infection within 7 days (Fig 4B). In contrast, BALB/c^TFE3-/-^ mice displayed markedly less weight loss compared to BALB/c^TFE3+/+^ mice, with three out of five BALB/c^TFE3-/-^ mice surviving up to 14 days post-infection, indicative of partial rescue from lethal AIV infection. Moreover, *NP* mRNA and *HA* mRNA levels in the lungs were significantly lower in BALB/c^TFE3-/-^ mice compared to BALB/c^TFE3+/+^ mice (Fig 4C). Additionally, mRNA levels of pro-inflammatory cytokines including *IL-6*, *TNF-α*, *IL-1β*, and *IL-8* were significantly reduced in BALB/c^TFE3-/-^ mice relative to BALB/c^TFE3+/+^ mice (Fig 4D), suggesting a critical role of TFE3 in viral replication and infection.

### TFE3 promotes the expression of glycosylation enzymes to facilitate AIV replication

**Fig 5:**
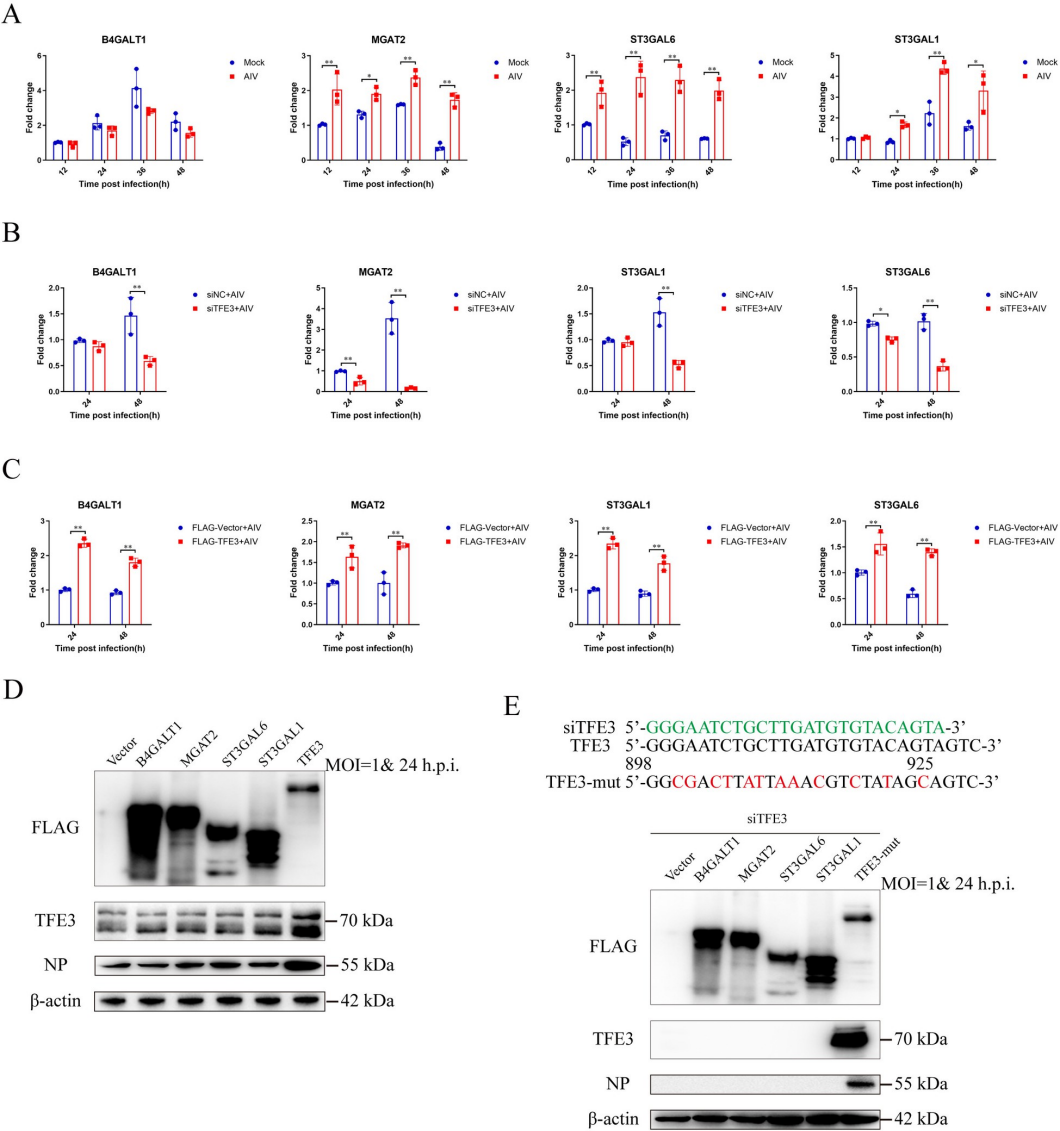
Effect of TFE3 on expression of glycosylation enzymes and AIV replication. (A) AIV infection activated transcription of N-glycosylation enzymes. A549 cells were infected with AIV at 1 MOI. At 12, 24, 36, and 48 h post-infection, the cells were harvested, and total RNA was extracted for RT-qPCR analyses. Effects of TFE3 silencing (B) or overexpression (C) on the regulation of N-glycosylation enzymes following AIV infection. A549 cells were transfected with siTFE3 or FLAG-TFE3 before infection with AIV. At 24 and 48 h post-infection, total RNA was extracted for RT-qPCR analyses. (D) Function of N-glycosylation enzymes on the replication of AIV. A549 cells were transfected with FLAG-B4GALT1, FLAG-MGAT2, FLAG-ST3GAL6, FLAG-ST3GAL1, and FLAG-TFE3 before AIV infection. At 24 h post-infection, cell lysates were subjected to western blotting, and the membranes were incubated with an anti-FLAG antibody (to confirm the expression of transfected constructs) and further incubated with antibodies against NP. β-actin was used as an internal standard. Protein bands were quantified using ImageJ. (E) Role of N-glycosylation enzymes in AIV replication after TFE3 knockdown. A549 cells were transfected with FLAG-B4GALT1, FLAG-MGAT2, FLAG-ST3GAL6, FLAG-ST3GAL1, and FLAG-mutTFE3 before AIV infection. At 24 h post-infection, cell lysates were incubated with antibodies against FLAG (for expression confirmation) and TFE3 (for interference confirmation), followed by incubation with antibodies against NP. β-actin was used as an internal standard. Error bars represent SD of the means from three independent experiments (*p<0.05, **p<0.01).

During GAS, TFE3 undergoes dephosphorylation and translocation into the nucleus, consequently initiating the transcription of genes associated with Golgi structural proteins, N-glycosylation enzymes, and vesicular transport components. To investigate the involvement of TFE3 in AIV replication, we assessed the expression levels of enzymes participating in N-glycosylation, including type 2 lactosamine alpha-2,3-sialyltransferase (*ST3GAL6*), CMP-N-acetylneuraminate-beta-galactosamide-alpha-2,3-sialyltransferase 1 (*ST3GAL1*), beta-1,4-galactosyltransferase 1 (*B4GALT1*), and alpha-1,6-mannosyl-glycoprotein 2-beta-N-acetylglucosaminyltransferase (*MGAT2*). Compared to mock-infected cells, AIV infection significantly upregulated the expression of ST3GAL6 and MGAT2 genes at 12-, 24-, 36-, and 48-hours post-infection, and ST3GAL1 gene at 24-, 36-, and 48-hours post-infection, while B4GALT1 expression remained largely unchanged (Fig 5A).

To further elucidate the regulatory role of TFE3 on N-glycosylation enzymes, we examined their mRNA levels following TFE3 manipulation. TFE3 silencing markedly decreased the mRNA levels of *ST3GAL6*, *ST3GAL1*, *B4GALT1*, and *MGAT2* post-AIV infection (Fig 5B), whereas TFE3 overexpression exhibited the opposite effect (Fig 5C).

Moreover, overexpression of *ST3GAL6*, *ST3GAL1*, and *MGAT2* led to increased NP protein levels following AIV infection in A549 cells (Figs 5D and S7A). However, upon TFE3 silencing, NP protein expression was undetectable even when N-glycosylation enzymes were overexpressed (Fig 5E). These findings indicate that TFE3 promotes the expression of N-glycosylation enzymes to facilitate viral replication.

### TFE3 upregulates vATPase and Rab7 expression to benefit endosome acidification to facilitate AIV replication

**Fig 6:**
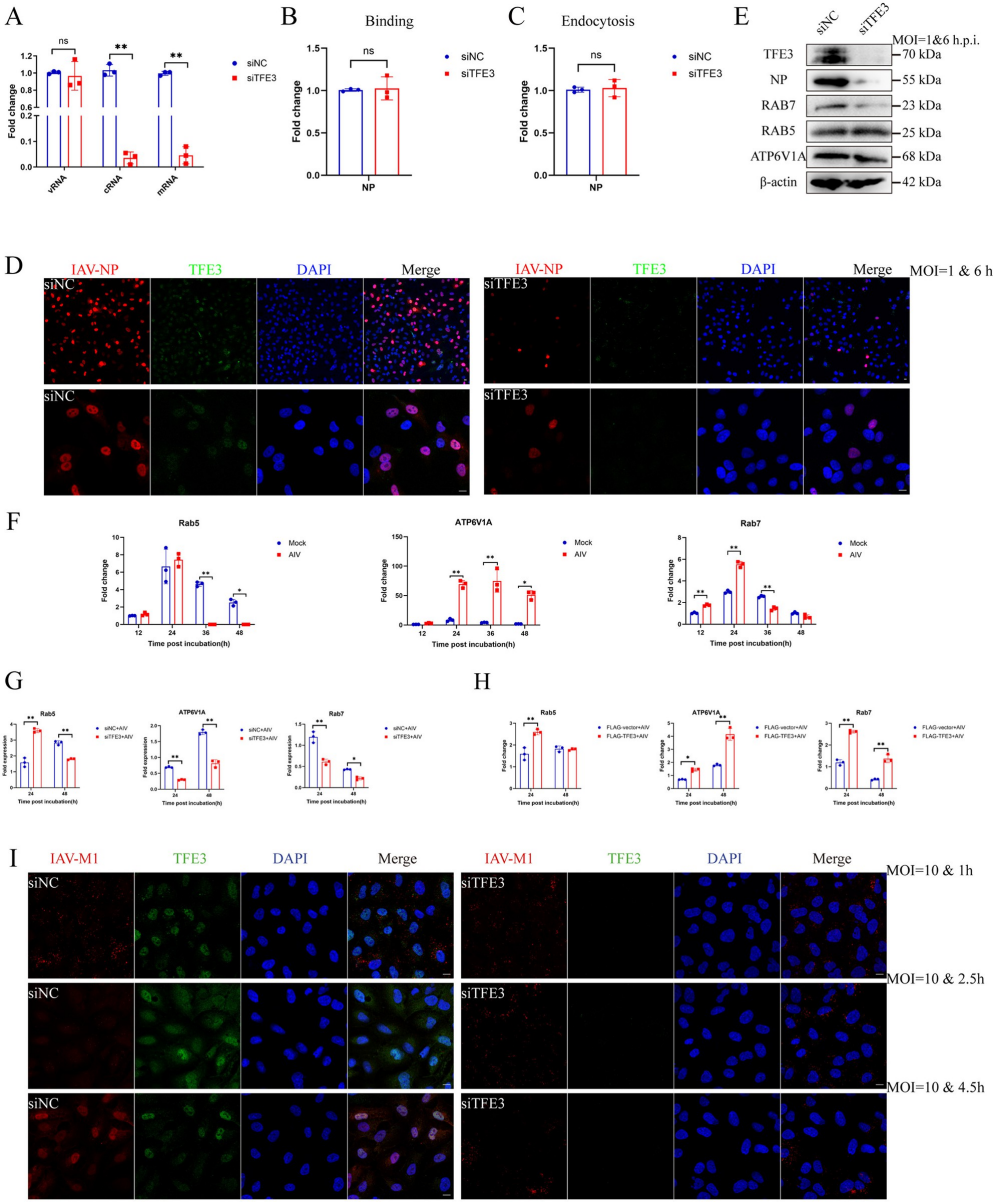
Effect of TFE3 on endosome acidification and AIV replication. (A) mRNA, vRNA, and cRNA levels of the NP gene after TFE3 knockdown. A549 cells were transfected with siTFE3 before AIV infection. mRNA, vRNA, and cRNA were reverse-transcribed and quantified using RT-qPCR. (B) Binding or (C) Endocytosis capacity of AIV after TFE3 knockdown. A549 cells were transfected with siTFE3 before AIV infection. Expression of NP was quantified using RT-qPCR. (D) NP nuclear translocation after TFE3 knockdown. A549 cells were transfected with siTFE3 before AIV infection. A549 cells were infected with AIV at 1 MOI, fixed at 6 h post-infection on slides, and incubated with antibodies against IAV-NP and TFE3 and subjected to DAPI staining. Cells were visualized using a Zeiss confocal fluorescence microscope LSM880 (IAV-NP, red; TFE3, green; DAPI, blue). Scale bar = 10 μm. (E) Expression of proteins related to the endosome. A549 cells were infected with AIV at 1 MOI, cell lysates were then prepared at 6 h post-infection and incubated with antibodies against IAV-NP, RAB5, RAB7, and ATP6V1A at 4°C overnight. β-actin was used as an internal standard. Bands were visualized using a chemiluminescence imaging analysis system after incubation with peroxidase-conjugated secondary antibodies. (F) AIV infection activated transcription of endosome-related genes. A549 cells were infected with AIV at 1 MOI. At 12, 24, 36, and 48 h post-infection, the cells were harvested, and total RNA was extracted for RT-qPCR analyses. (G) Effects of TFE3 knockdown or (H) TFE3 overexpression on the regulation of endosome-related genes following AIV infection. A549 cells were transfected with siTFE3 or FLAG-TFE3 before infection with AIV. At 24 and 48 h post-infection, total RNA was extracted for RT-qPCR analyses. (I) M1 distribution after TFE3 knockdown. A549 cells were transfected with siTFE3 before AIV infection. A549 cells were infected with AIV at 10 MOI, fixed at 1 h, 2.5 h, and 4.5 h post-infection on slides, and incubated with antibodies against IAV-M1 and TFE3 and subjected to DAPI staining. Cells were visualized using a Zeiss confocal fluorescence microscope LSM880 (IAV-M1, red; TFE3, green; DAPI, blue). Scale bar = 10 μm. Error bars represent SD of the means of data from three independent experiments (*p<0.05, **p<0.01).

To ascertain the involvement of TFE3 in viral replication, we analyzed the levels of viral RNA (vRNA), messenger RNA (mRNA), and complementary RNA (cRNA) of the AIV NP gene. While no significant alterations were observed in vRNA levels, TFE3 knockdown resulted in a notable decrease in mRNA and cRNA levels (Fig 6A). Subsequently, we investigated the binding and endocytosis capacity of AIV following TFE3 knockdown. Surprisingly, no significant changes were observed in the binding and endocytosis capacity of AIV post-TFE3 knockdown (Fig 6B and 6C).

Furthermore, we examined the nuclear translocation of NP after TFE3 knockdown. Remarkably, while numerous NP proteins were detected in the nucleus in the siNC group, only limited NP proteins were observed in the nucleus in the siTFE3 group (Fig 6D). These findings suggest that TFE3’s role in avian influenza viral replication may occur before vRNP nuclear translocation and after viral endocytosis.

To further explore this mechanism, we analyzed early endosomes, Vacuolar-type ATPase (vATPase), and late endosomes. Our results revealed that TFE3 knockdown led to decreased expression of the late endosome marker Rab7 and the vATPase component ATP6V1A, indicating impaired endosome acidification (Figs 6E and S7B). Moreover, compared to mock-infected cells, AIV infection significantly downregulated *Rab5* gene expression at 36- and 48-hours post-infection, while significantly upregulating *ATP6V1A* gene expression at 24-, 36- and 48-hours post-infection (Fig 6F). Additionally, AIV infection significantly upregulated *Rab7* gene expression at 12-and 24-hours post-infection but downregulated it at 36-hours post-infection. Our results also demonstrated that TFE3 knockdown led to decreased expression of the late endosome marker Rab7 and the vATPase component ATP6V1A (Fig 6G). Conversely, TFE3 overexpression resulted in increased expression of these markers (Fig 6H), indicating TFE3 may function through endosome acidification.

Given that pH levels decrease progressively from early to late endosomes, and AIV HA protein requires a relatively low pH for conformational changes necessary for membrane fusion or uncoating during initial infection, we next examined the distribution of the M1 protein over the course of AIV infection. Interestingly, while AIV M1 protein exhibited a transition from an aggregation morphology to a diffuse morphology to nuclear translocation morphology over time in the siNC group, indicative of normal AIV infection, M1 protein maintained an aggregation morphology throughout infection time points in the siTFE3 group, suggesting AIV particles were trapped in the endosome (Fig 6I). Consequently, our findings suggest that TFE3 promotes the expression of vATPase and Rab7 to facilitate endosome acidification, thereby enhancing AIV replication.

## Discussion

In mammalian cells, the Golgi apparatus is a crucial membrane-bound organelle typically positioned near the nucleus. Its role involves receiving proteins and lipids from the endoplasmic reticulum (ER) via the cis-cisternae and exporting them to various intracellular destinations, including endosomes [24–27], lysosomes [28], plasma membranes [29,30], and outside the cell [31]. The Golgi apparatus operates in tandem with the trans-Golgi network to facilitate these intricate processes. The Golgi apparatus stress (GAS) response represents a self-regulating mechanism that heightens Golgi function in response to insufficiencies [10]. Despite its importance, the relationship between GAS and viral infections remains poorly understood. However, emerging research has begun to shed light on this topic. For instance, Ganesan et al. discovered that hepatitis B virus (HBV) infection independently triggers Golgi fragmentation, which hinders HBV peptide-MHC class I complex presentation, potentially aiding in viral evasion of cytotoxic T-lymphocyte recognition and promoting viral persistence in hepatocytes [32]. Similarly, Daussy et al. demonstrated that hepatitis C virus (HCV) infection induces Golgi fragmentation through immunity-related GTPase family M (IRGM), facilitating viral replication [33]. Moreover, Hansen et al. elucidated the role of IRGM in connecting intracellular membrane remodeling via autophagy to Golgi fragmentation, contributing to HCV replication [34]. Additionally, HSV-1 has been found to exploit GM130-mediated GAS to breach the blood-brain barrier, enhancing its infectivity [35]. Furthermore, Cortese et al. revealed that SARS-CoV-2 induces morphological alterations and fragmentation of the Golgi apparatus, affecting the secretory route and potentially facilitating viral propagation [36]. Of particular relevance to our study, Pandey highlighted that influenza virus infection leads to dispersed trans-Golgi network (TGN), triggering NLRP3 inflammasome activation. Further investigations implicated specific residues of the M2 protein of influenza virus as pivotal in this process [37]. Additionally, Viettri demonstrated that infection with dengue and Zika viruses induces Golgi complex dilatation and translocation of TFE3, indicating Golgi apparatus stress due to heightened demand imposed by virion and NS1 processing and secretion [38]. Our study founded that AIV infection induces GAS, resulting in Golgi apparatus fragmentation in A549 cells. Specially, AIV infection selectively activates the TFE3 pathway within the GAS response, while the HSP47 and CREB3-ARF4 pathways remain largely unaffected or minimally activated.

Fragmentation of the Golgi apparatus poses significant challenges to vesicle transport [39] and protein modification processes [40,41], resulting in aberrant protein modification. In our study, we observed Golgi apparatus fragmentation induced by AIV infection, particularly during the middle and later stages of the infection process. At this juncture, the virus had already replicated sufficiently, and the demand for large-scale protein synthesis diminished for its survival. Interestingly, we noted a paradoxical observation wherein *GM130* mRNA levels increased while its protein levels decreased following AIV infection. This discrepancy could potentially be attributed to the compensatory response initiated by TFE3 dephosphorylation and nuclear transportation. This cascade triggers the expression of various genes, including GM130, in an attempt to alleviate Golgi apparatus stress. However, prolonged Golgi apparatus stress may eventually lead to its dispersion, resulting GM130 protein degradation. A previous study has reported the association of 26S proteasomes with the cytosolic surface of Golgi membranes, facilitating Golgi Apparatus-Related Degradation (GARD) and subsequent degradation of GM130 in response to Golgi stress [42].

The HSP47 pathway is recognized for its anti-apoptotic role during Golgi apparatus stress [13]. Contrary to expectations, our study revealed that the HSP47 pathway limitedly activated following AIV infection. Interestingly, when HSP47 was overexpressed, it significantly reduced apoptosis in A549 cells subsequent to AIV infection, whereas its silencing had the opposite effect, as depicted in S8 Fig. Examining the viral growth curve, we observed a slight increase in virus titers at 12 hours post-infection with HSP47 overexpression. Conversely, a moderate decrease in titers was evident upon HSP47 silencing. These findings suggest that the HSP47 pathway might exert its anti-apoptotic function, particularly during the early stages of efficient viral replication, as part of the GAS response triggered by AIV infection.

TFE3, a member of the basic helix-loop helix leucine zipper (bHLH-LZ) transcription factor family [43], is implicated in various cellular processes, including cell starvation, ER stress, mitochondrial damage, pathogen infection, lysosomal stress, and Golgi apparatus stress [10,11,44,45]. Notably, TFE3 overexpression in mammalian cells stimulates the transcription of the Golgi Apparatus Stress Response Element (GASE), characterized by the consensus sequence ACGTGGC, thereby orchestrating the transcriptional activation during Golgi apparatus stress [11,45]. Under normal conditions, TFE3 undergoes dephosphorylation at Ser108 and translocates to the nucleus, where it triggers the transcriptional activation of GASE. The target genes regulated by the TFE3 pathway encompass Golgi structural proteins (such as *GCP60*, *GM130*, and *giantin*), N-glycosylation enzymes (including *ST3GAL1*, *ST3GAL6*, *FUT1*, *B3GAT2*, and *UAP1L1*), and vesicular transport components [11]. In our investigation, we observed activation of the TFE3 pathway subsequent to AIV infection. Notably, TFE3 overexpression substantially heightened AIV replication, while its silencing exhibited the converse effect. Furthermore, TFE3 knockout attenuated AIV replication and augmented the survival rate of mice. These findings underscore the pivotal role of the TFE3 pathway in mediating the Golgi apparatus stress response triggered by AIV infection. The upregulation of N-glycosylation enzymes subsequent to TFE3 pathway activation may contribute to the modification of viral envelope glycoproteins, thereby facilitating AIV replication.

Endocytosis serves as a crucial mechanism for cells to internalize nutrients and external signals, facilitating the completion of various cellular processes. Viruses like AIV can exploit endocytosis to invade host cells [46]. Upon internalization, the viral genome resides within early endosomes, which gradually acidify to form late endosomes. At this juncture, the viral hemagglutinin protein undergoes a conformational change, triggering the release of fusion peptides. These peptides facilitate fusion between the viral membrane and the endosomal membrane, enabling the release of the viral genome into the host cell cytoplasm [46]. Consequently, the acidification of endosomes plays a crucial role in AIV replication. Our study unveiled that TFE3 not only upregulates the expression of N-glycosylation enzymes but also regulates key components such as v-ATPase and Rab7, thereby promoting AIV replication. Upon TFE3 knockdown, AIV particles become trapped within endosomes, impeding their uncoating process. Furthermore, our investigation extended to other viruses, including NDV, VSV, and HSV, revealing that TFE3 knockdown also impedes their replication. These findings suggest that TFE3 inhibition could serve as a promising strategy for developing broad-spectrum antiviral drugs, particularly against viruses reliant on endosome acidification or Golgi apparatus protein modification for their life cycle.

**Fig 7:**
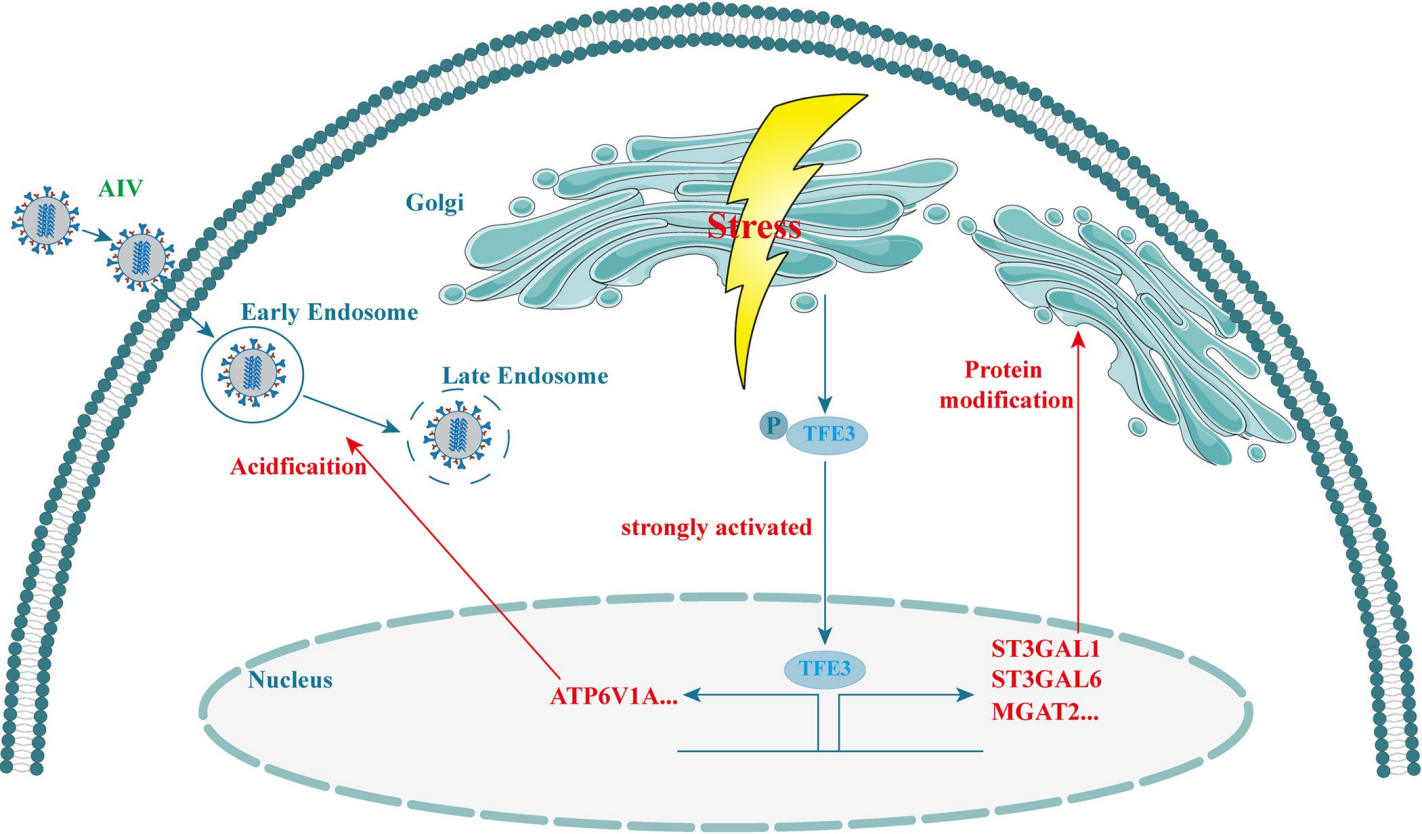
Schematic of Golgi apparatus stress response pathway activated by AIV infection. AIV infection resulted in Golgi apparatus stress (GAS), characterized by swelling and fragmentation of the Golgi apparatus. During GAS response, the TFE3 pathway exhibited robust activation. The activation of the TFE3 pathway within the GAS response promoted endosome acidification and increased the transcription of glycosylation enzymes, thereby facilitating AIV replication.

In summary, our study elucidated that AIV infection induces Golgi apparatus stress (GAS) via the TFE3 pathway in A549 cells, facilitating endosome acidification and activating N-glycosylation enzymes to enhance virus replication (Fig 7). These discoveries contribute to a deeper comprehension of AIV infection dynamics and the consequential organelle stress that fosters AIV propagation.

## Material and methods

### Ethics statement

Six-week-old female BALB/c mice were purchased from Experimental Animal Center of Yangzhou University (Yangzhou, China). All animal experiments were performed in strict compliance with the Guidelines of Laboratory Animal Welfare and Ethics of Jiangsu Administrative Committee for Laboratory Animals and were approved by the Jiangsu Administrative Committee for Laboratory Animals (Permission Number: SYXK-SU-2017-0044). The mice were monitored daily for clinical signs of morbidity and mortality for up to 14 days post-infection. When the animals lost 25% or more of their initial body weight during the study, they were scored dead and euthanized under excess isoflurane anesthesia according to the institutional guidelines. All efforts were made to minimize suffering.

### Cells, viruses, plasmids, and antibodies

The adenocarcinoma human alveolar basal epithelial cell line A549 (ATCC, Manassas, VA, USA) was cultured in Ham’s F-12K (Kaighn’s) medium (21127–022, ThermoFisher Scientific, Waltham, MA, USA) supplemented with 10% fetal bovine serum (10100, Gibco, Grand Island, NY, USA) and 1% Glutamax (35050, Thermo Fisher, Grand Island, NY, USA) at 37°C with 5% CO_2_. The H5 subtype AIV strains A/Mallard/Huadong/S/2005 (named AIV in this study), A/California/04/09 (CA04, H1N1), A/Puerto Rico/8/1934 (PR8, H1N1), A/Chicken/Taixing/10/2010 (TX, H9N2), and A/Chicken/Guangdong/04/2017 (GD, H7N9) were propagated in allantoic cavities of 10-day-old specific pathogen-free embryonic chicken eggs. Antibodies against TFE3 (14779), GM130 (12480), and β-actin (3700) were purchased from Cell Signaling Technology (Danvers, MA, USA) and those against HSP47 (ab109117), ARF4 (ab171746), IAV-NP (ab20343), and CREB3 (ab180119) from Abcam (Cambridge, UK). Monoclonal antibodies against IAV-M1 (sc-57881), VSV (sc-365019) and HSV (sc-57863) was purchased from Santa Cruz Biotechnology. Monoclonal antibody against NDV-NP was obtained from Chan Ding’s lab and Monoclonal antibody against IAV-HA(H5) was obtained from Daxin Peng’s lab. 4-phenylbutyric acid (5 μM), Monensin (10 μM), BFA (10 μM) were purchased from Selleck (Shanghai, China). *HSP47*-, *CREB3*-, and *TFE3-*specific siRNAs were purchased from GenePharma (Shanghai, China). The silencing oligonucleotides sequences used were as follows: HSP47 (5′-GCAGCAAGCAGCACUACAA-3′), CREB3 (5′-CGACUGGGAAGUAGAUGAUUUGC-3′), and TFE3 (5′- GGGAAUCUGCUUGAUGUGUACAGUA -3′)

### Virus infection and determination of cytokine levels

Monolayer A549 cells were infected with each tested virus in F-12K at 1 MOI for 1 h, washed to remove unbound viruses, and incubated in F-12K at 37°C. The mRNA levels of *ARF4*, *HSP47*, *GM130*, *TFE3*, *B3GAT3*, *HS6ST1*, *GALNT5*, *GALNT8*, *GALNT18*, *HA*, *NP*, *TNF-α*, *IL-1β*, *IL-6*, *IL-8*, *MGAT2*, *B4GALT1*, *ST3GAL1*, *ST3GAL6*, *Rab5*, *Rab7* and *ATP6V1A* were determined using reverse transcription-quantitative polymerase chain reaction (RT-qPCR). Briefly, total RNA was isolated using TRIzol (15596–026, Thermo Fisher Scientific), and 2 μg of RNA was reverse-transcribed to cDNA using the HiScript II 1st Strand cDNA Synthesis Kit +gDNA wiper kit (R212, Vazyme Biotech, Nanjing, China), according to the manufacturer’s protocol. qPCR was performed using SYBR Green (A660A, Promega, Madison, WI, USA), and the housekeeping gene *GAPDH* was used as an internal standard. The primer sequences used for amplification are listed in S1 Table.

### Determination of viral mRNA, vRNA, and cRNA levels

Monolayer A549 cells were infected with each tested virus in F-12K at 1 MOI for 1 h, washed with PBS to remove unbound viruses, and incubated in F-12K at 37°C for 4 h. Total RNA was isolated, and mRNA, vRNA, and cRNA were reverse-transcribed using suitable primers (mRNA: 5’-CCAGATCGTTCGAGTCGT-TTTTTTTTTTTTTTTTCTTTAATTGTC-3’; vRNA: 5’-GGCCGTCATGGTGGCGAATGAATGGACGGAGAACAAGGATTGC-3’; cRNA: 5’-GCTAGCTTCAGCTAGGCATCAGTAGAAACAAGGGTATTTTTCTTT-3’) according to Kawakami [47]. qPCR was performed using SYBR Green, and *GAPDH* was used as an internal standard.

### Western blot analysis

A549 cells were transfected with siRNAs and then infected with virus. The cells were washed and lysed in RIPA buffer (P0013B; Beyotime Biotechnology, Shanghai, China) containing a protease inhibitor cocktail (B14001; Bimake, Houston, TX, USA). The lysates were denatured, subjected to sodium dodecyl sulfate-polyacrylamide gel electrophoresis, and transferred to nitrocellulose membranes (1060002, GE Healthcare, Little Chalfont, UK). The membranes were then blocked with non-fat milk and incubated with respective primary antibodies overnight at 4°C, followed by incubation with horseradish peroxidase-conjugated secondary antibodies (111-035-003 and 115-035-003, Jackson ImmunoResearch Laboratories, West Grove, PA, USA) for 1 h at room temperature. Protein bands were visualized using SuperSignal West Pico PLUS Chemiluminescence Substrate (34580, Thermo Fisher Scientific) and quantified using ImageJ (NIH, Bethesda, MD, USA). Mock-transfected cells were used as the control.

### Immunofluorescence experiment

Monolayer A549 cells grown on glass coverslips were infected with virus, or infected with virus and drug treatment (4-phenylbutyric acid, 5 μM; Monensin, 10 μM; BFA, 10 μM) for detection at indicated time points. The cells were then washed and fixed in 4% formaldehyde, permeabilized with PBS containing 0.5% Triton X-100 for 10 min and incubated in PBS containing 3% bovine serum albumin. The cells were then incubated with primary antibodies for 1 h at 37°C, washed three times with PBS, incubated with fluorescence-conjugated secondary antibodies (A32731 and A11032, Thermo Fisher Scientific) for 1 h, and then washed three times before staining with DAPI (62247, Thermo Fisher Scientific) at 1:5000 dilution for 10 min. Finally, coverslips were mounted on glass slides and visualized using a Zeiss confocal fluorescence microscope LSM880 (Oberkochen, Germany).

### RNA interference

RNA interference was used to knock down *CREB3*, *HSP47*, and *TFE3* genes. A549 cells were cultured in a 6-well plate to 40–50% confluence and transfected with siRNAs using Lipofectamine 2000 (11668, Thermo Fisher Scientific) as described by the manufacturer. siRNA and Lipofectamine 2000 were diluted in 50 μL of serum-free Opti-MEM medium (31985, Thermo Fisher Scientific), incubated, mixed, and added to each well. At 4 h post-transfection, the cells were washed three times with PBS and incubated for additional 36 h prior to viral infection.

### Cell apoptosis

Monolayer A549 cells were infected with virus, with siRNA treatment for the indicated time points. The cells were washed with PBS, harvested, washed again with pre-cold PBS, and resuspended in an annexin binding buffer (V13245, Thermo Fisher Scientific). PI and Alexa Fluor 488 annexin V (V13245, Thermo Fisher Scientific) were added to the cell suspension. After incubation at room temperature for 15 min, the number of cells was determined using a cytometer.

### Determination of virus growth

Monolayer A549 cells were transfected with siRNAs or eukaryotic expression plasmids and then infected with virus s at a dose of 1 MOI in F-12K for 1 h. The supernatant samples were collected to determine the TCID_50_ values in CEF cells at 12, 24, 36 and 48 h.p.i.. For TCID_50_ evaluation, each sample was serially diluted 10-fold from 10^−1^ to 10^−9^, and each dilution (10^−1^–10^−9^) was inoculated into MDCK cells. The TCID_50_/mL were calculated as previously described.

### Virus binding detection

Monolayer A549 cells were transfected with siRNAs and then infected with virus s at a dose of 10 MOI in F-12K for 1 h at 4°C. Cells were washed with PBS to remove uncombined viruses and then total cell RNA were extracted for virus quantification.

### Virus endocytosis detection

Monolayer A549 cells were transfected with siRNAs and then infected with virus s at a dose of 10 MOI in F-12K for 1 h at 4°C. Cells were washed with PBS to remove uncombined viruses and incubated for additional 1 h at 37°C. Cells were washed with PBS-HCL (pH = 2.0) to remove un-internalized viruses and then total cell RNA were extracted for virus quantification.

### NP nuclear translocation detection

Monolayer A549 cells were transfected with siRNAs and then infected with virus s at a dose of 1 MOI in F-12K for 1 h at 4°C. Cells were washed with PBS to remove uncombined viruses and incubated for additional 6 h at 37°C. Cells were fixed and sent for immunofluorescence experiment to detect NP nuclear translocation.

### M1 distribution detection

M1 distribution detection was according to Zou’s study [48]. Briefly, Monolayer A549 cells were transfected with siRNAs and then infected with virus s at a dose of 1 MOI in F-12K for 1 h at 4°C. Cells were washed with PBS to remove uncombined viruses and incubated for additional 1 h, 2.5 h and 4.5 h at 37°C. Cells were fixed and sent for immunofluorescence experiment to detect M1 distribution.

### Mice experiments

Each eleven 6-week-old BALB/c (BALB/c^TFE3+/+^, n = 11, Experimental Animal Center of Yangzhou University, Yangzhou, China) and BALB/c *TFE3* knockout mice (BALB/c^TFE3-/-^, n = 11, GemPharmatech Corporation, Nanjing, China) were infected intranasally with AIV at 10^6^ EID_50_ in 50 μL PBS. After infection, BALB/c^TFE3+/+^ (n = 5) and BALB/c^TFE3-/-^ (n = 5) mice were weighed individually, and monitored for signs of illness and mortality daily for 2 weeks to measure the weight change and death rate; each three mice (BALB/c^TFE3+/+^, n = 3; and BALB/c^TFE3-/-^, n = 3) were euthanized on days 3 and 5 post-infection respectively, and their lungs were collected for RNA extraction to measure the viral titer and determine the expression of inflammatory genes.

### Statistical analysis

All data were expressed as the mean ± standard deviation. Statistical significance was determined using one-way analysis of variance.

## Supporting information

S1 Fig Golgi apparatus morphology in A549 cells. (A) AIV infection-induced collapse of the Golgi apparatus at different time points. A549 cells were infected with AIV at 1 MOI, fixed on slides at 12, 24, 36, and 48 h post-infection, and incubated with antibodies against IAV-NP, GM130, and DAPI. Scale bar = 10 μm. (B) Counts of AIV infection-induced collapse of the Golgi apparatus at different time points.
(PDF)

S2 Fig Confirmation of ER stress inhibition. A549 cells were infected with AIV at 1 MOI with or without 4-phenylbutyric acid, cell lysates were then prepared at 24 h post-infection and incubated with antibodies against IRE1α and pIRE1α at 4°C overnight. β-actin was used as an internal standard. Bands were visualized using a chemiluminescence imaging analysis system after incubation with peroxidase-conjugated secondary antibodies.
(PDF)

S3 Fig Golgi apparatus morphology in A549 cells infected by different subtypes of influenza viruses. Influenza A virus (IAV) infection-induced collapse of the Golgi apparatus (36 h). A549 cells were infected with different IAVs at 1 MOI, fixed at 36 h post-infection on slides, and incubated with antibodies against IAV-NP, GM130, and subjected to DAPI staining. Slides were visualized using a Zeiss confocal fluorescence microscope LSM880. Scale bar = 10 μm.
(PDF)

S4 Fig Grayscale of expression of proteins related to Golgi apparatus stress (GAS) response pathways following AIV infection. Analysis of protein levels related to GAS response pathways induced by AIV infection at different times (A) or MOIs (B). Protein bands were quantified using ImageJ.
(PDF)

S5 Fig TFE3 nuclear translocation in A549 cells infected by different subtypes of influenza viruses. (A) Influenza A virus (IAV) infection-induced collapse of the Golgi apparatus (36 h). A549 cells were infected with different IAVs at 1 MOI, fixed at 36 h post-infection on slides, and incubated with antibodies against IAV-NP, TFE3, and subjected to DAPI staining. Slides were visualized using a Zeiss confocal fluorescence microscope LSM880. Scale bar = 10 μm.
(PDF)

S6 Fig Effects of TFE3 silencing on virus replication. The cells were transfected with siTFE3 before AIV infection (A) or NDV, VSV, HSV infection (B). Cell lysates were incubated with antibodies against TFE3 to confirm RNA interference, and then incubated with antibodies against IAV HA, IAV M1 (A) or NDV-NP, VSV, HSV. β-actin was used as an internal standard.
(PDF)

S7 Fig **Expression of proteins related to protein modification (A) or endosome (B) following AIV infection.** Analysis of protein levels related to protein modification (A) or endosome (B). Protein bands were quantified using ImageJ.
(PDF)

S8 Fig Effect of HSP47 protein on apoptosis of AIV-infected A549 cells. Overexpression (A) or knockdown (B) of HSP47 affects apoptosis after AIV replication in A549 cells. A549 cells were transfected with FLAG-HSP47 or FLAG-vector before infection with tested viruses. The cells were harvested at 12, 24, 36, and 48 h post-infection and stained using PI and Alexa Fluor 488 annexin V. The extent of apoptosis was determined using flow cytometry. Error bars represent SD of the mean from three independent experiments (*p<0.05, **p< 0.01)
(PDF)

S1 Table Primers used in this study. (DOCX)
