# Supplementary figures and images for: H5 subtype avian influenza virus induces Golgi apparatus stress response via TFE3 pathway to promote virus replication
Source: PLoS Pathog. 2024 Dec 9;20(12):e1012748. doi: 10.1371/journal.ppat.1012748 (PMC11627363; doi:10.1371/journal.ppat.1012748)

S1 Fig

A

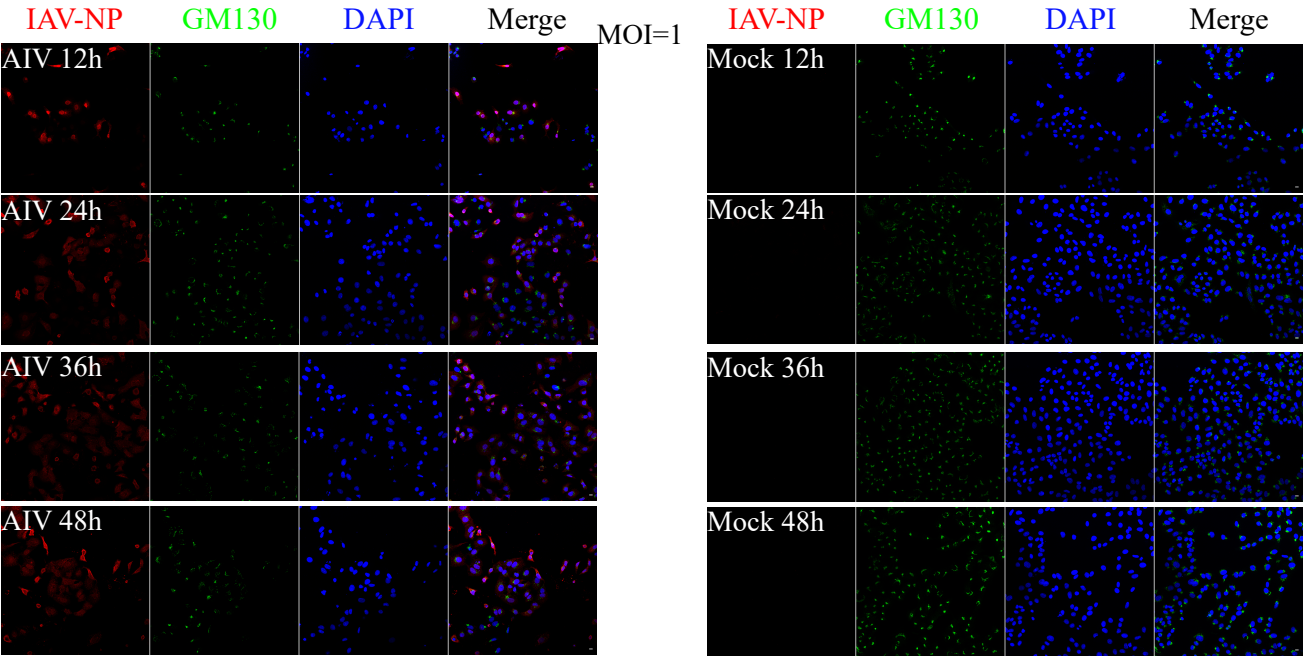

B

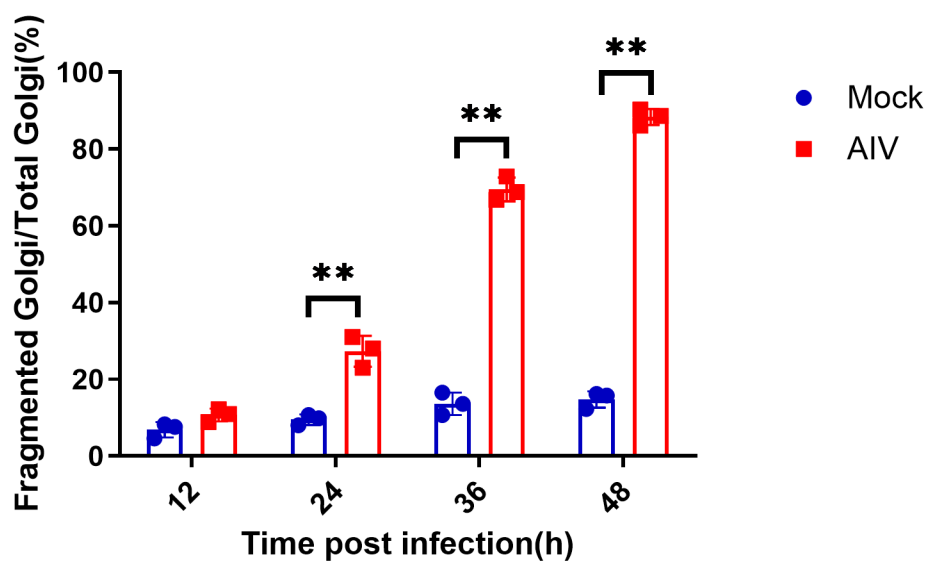

Supplement: S1 Fig — (A) AIV infection-induced collapse of the Golgi apparatus at different time points. A549 cells were infected with AIV at 1 MOI, fixed on slides at 12, 24, 36, and 48 h post-infection, and incubated with antibodies against IAV-NP, GM130, and DAPI. Scale bar = 10 μm. (B) Counts of AIV infection-induced collapse of the Golgi apparatus at different time points. (PDF) [file ppat.1012748.s001.pdf]

S2 Fig

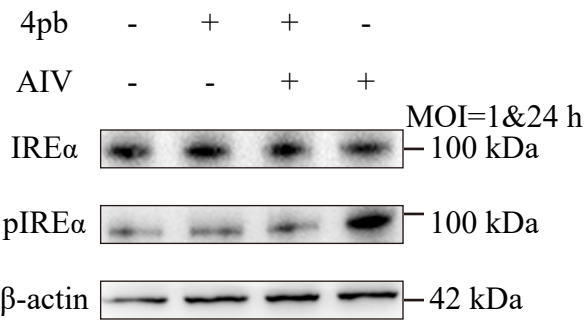

Supplement: S2 Fig — A549 cells were infected with AIV at 1 MOI with or without 4-phenylbutyric acid, cell lysates were then prepared at 24 h post-infection and incubated with antibodies against IRE1α and pIRE1α at 4°C overnight. β-actin was used as an internal standard. Bands were visualized using a chemiluminescence imaging analysis system after incubation with peroxidase-conjugated secondary antibodies. (PDF) [file ppat.1012748.s002.pdf]

S3 Fig

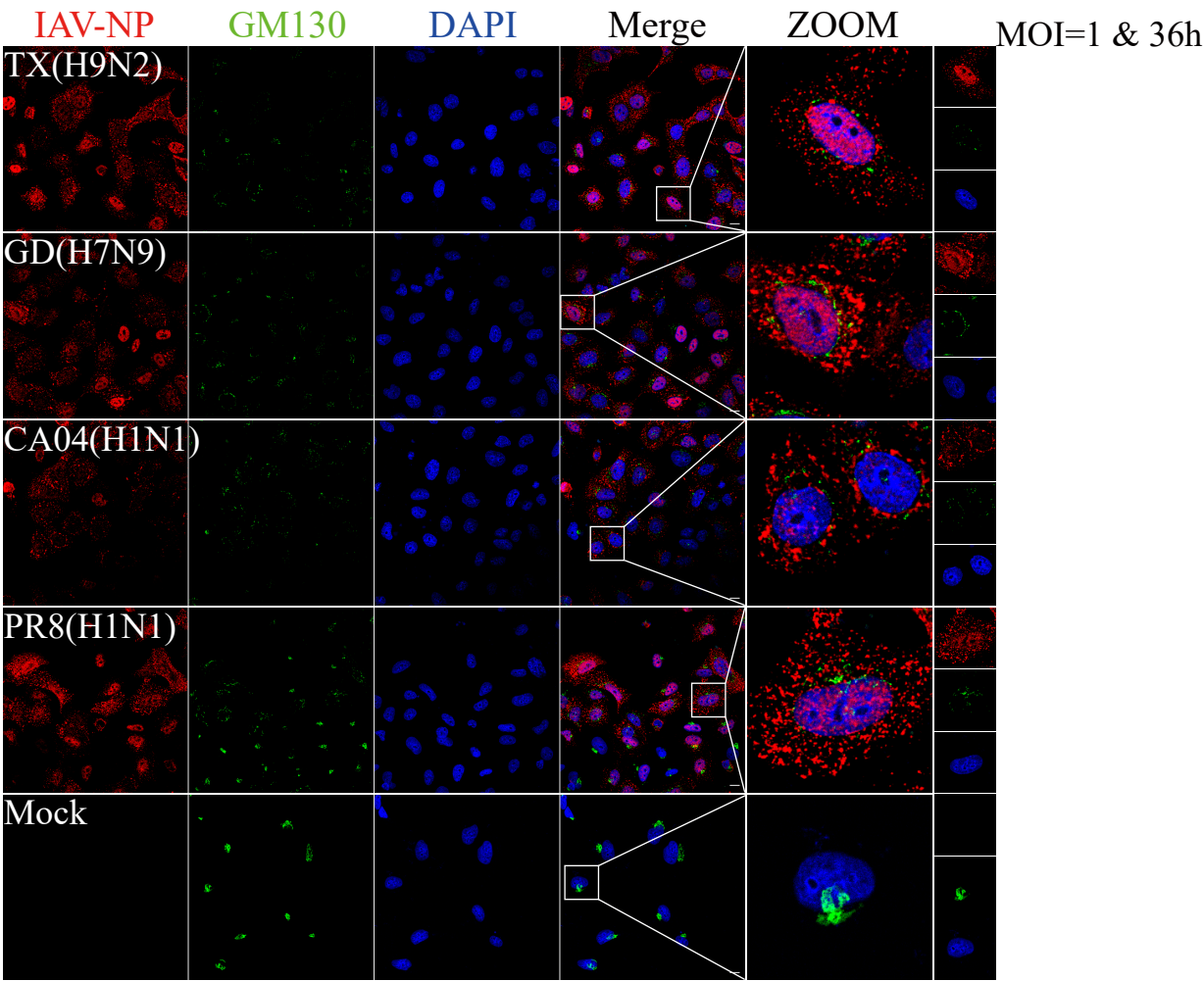

Supplement: S3 Fig — Influenza A virus (IAV) infection-induced collapse of the Golgi apparatus (36 h). A549 cells were infected with different IAVs at 1 MOI, fixed at 36 h post-infection on slides, and incubated with antibodies against IAV-NP, GM130, and subjected to DAPI staining. Slides were visualized using a Zeiss confocal fluorescence microscope LSM880. Scale bar = 10 μm. (PDF) [file ppat.1012748.s003.pdf]

S4 Fig

A

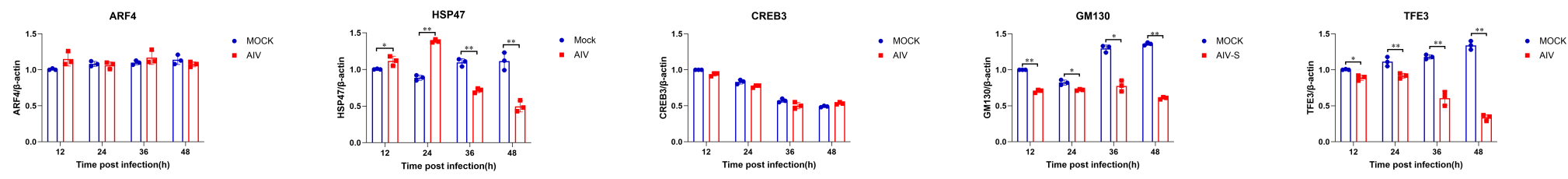

B

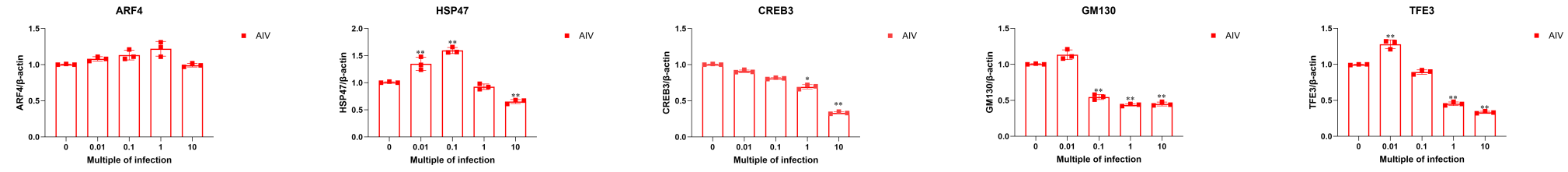

Supplement: S4 Fig — Analysis of protein levels related to GAS response pathways induced by AIV infection at different times (A) or MOIs (B). Protein bands were quantified using ImageJ. (PDF) [file ppat.1012748.s004.pdf]

S5 Fig

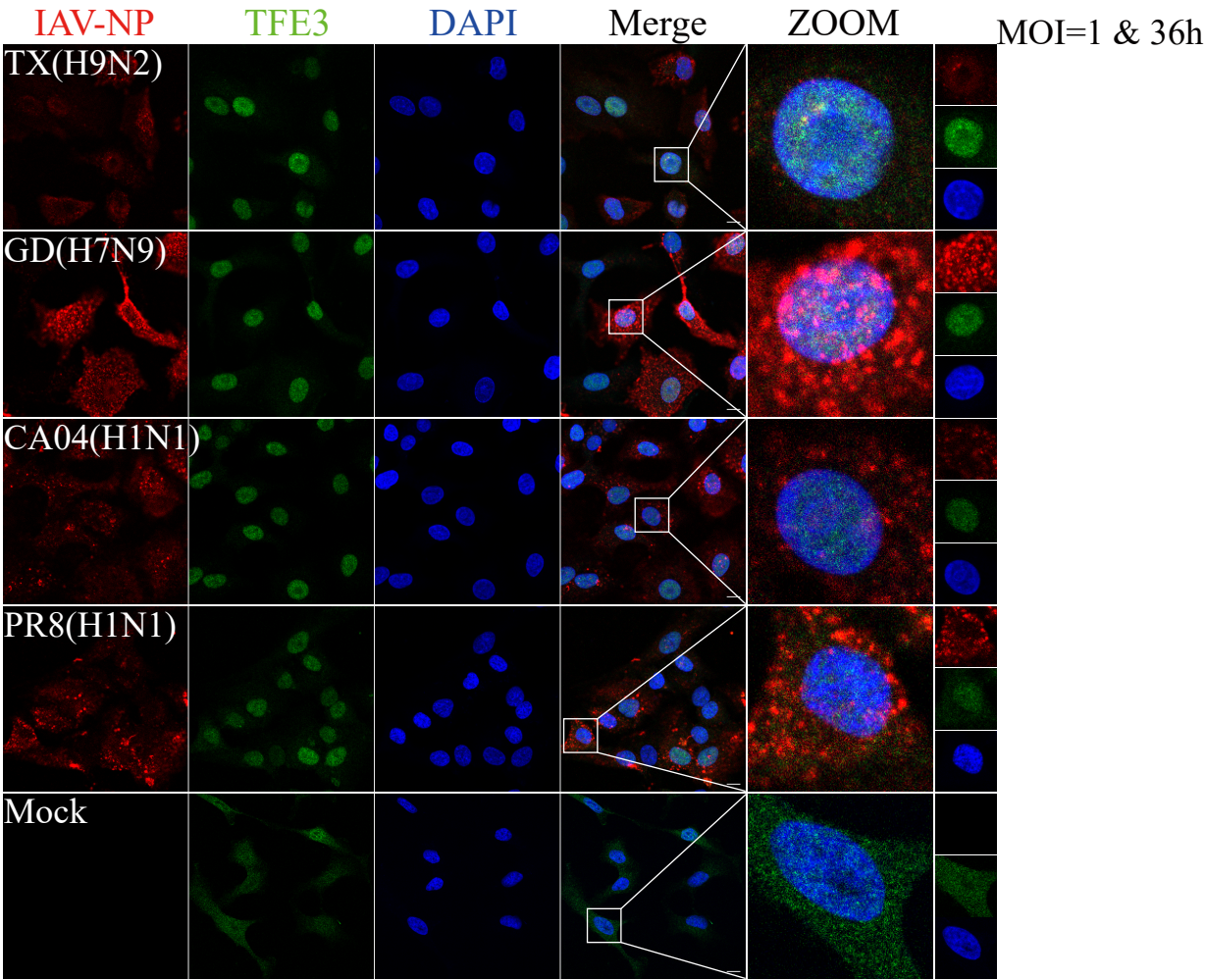

Supplement: S5 Fig — (A) Influenza A virus (IAV) infection-induced collapse of the Golgi apparatus (36 h). A549 cells were infected with different IAVs at 1 MOI, fixed at 36 h post-infection on slides, and incubated with antibodies against IAV-NP, TFE3, and subjected to DAPI staining. Slides were visualized using a Zeiss confocal fluorescence microscope LSM880. Scale bar = 10 μm. (PDF) [file ppat.1012748.s005.pdf]

S6 Fig

A

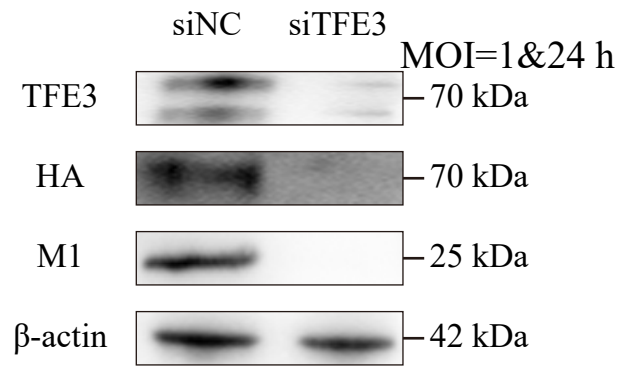

B

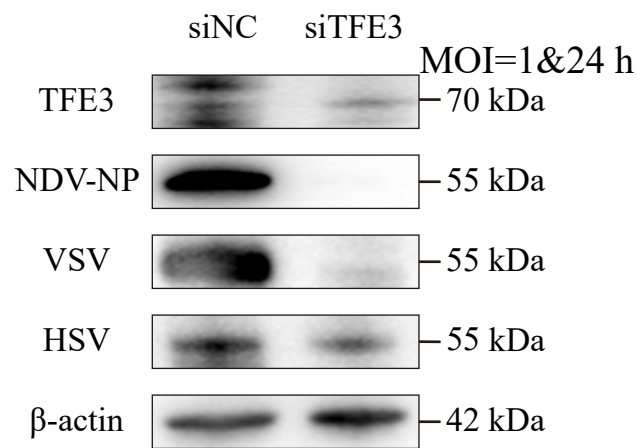

Supplement: S6 Fig — The cells were transfected with siTFE3 before AIV infection (A) or NDV, VSV, HSV infection (B). Cell lysates were incubated with antibodies against TFE3 to confirm RNA interference, and then incubated with antibodies against IAV HA, IAV M1 (A) or NDV-NP, VSV, HSV. β-actin was used as an internal standard. (PDF) [file ppat.1012748.s006.pdf]

S7 Fig

A

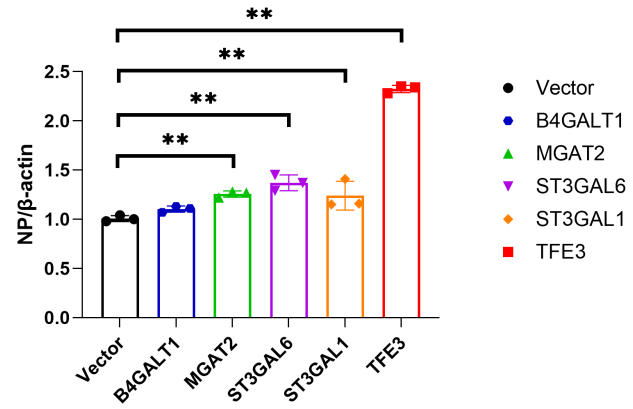

B

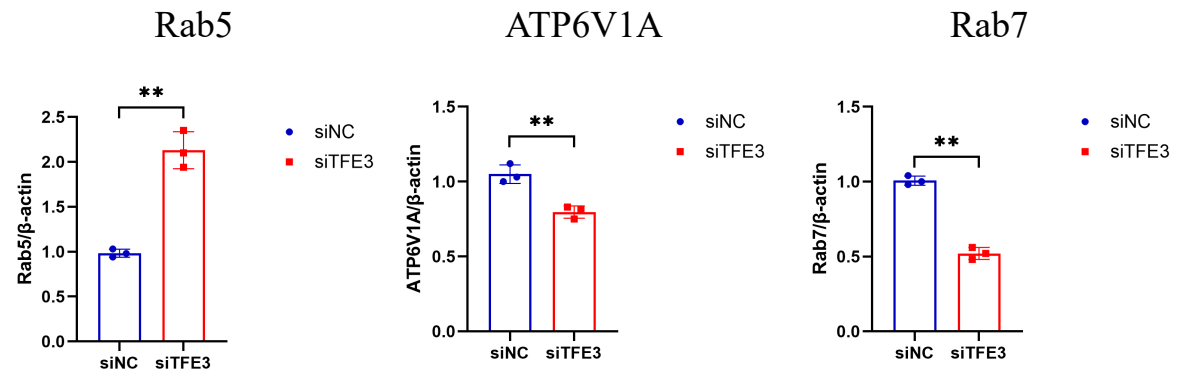

Supplement: S7 Fig — Expression of proteins related to protein modification (A) or endosome (B) following AIV infection. Analysis of protein levels related to protein modification (A) or endosome (B). Protein bands were quantified using ImageJ. (PDF) [file ppat.1012748.s007.pdf]

S8 Fig

A

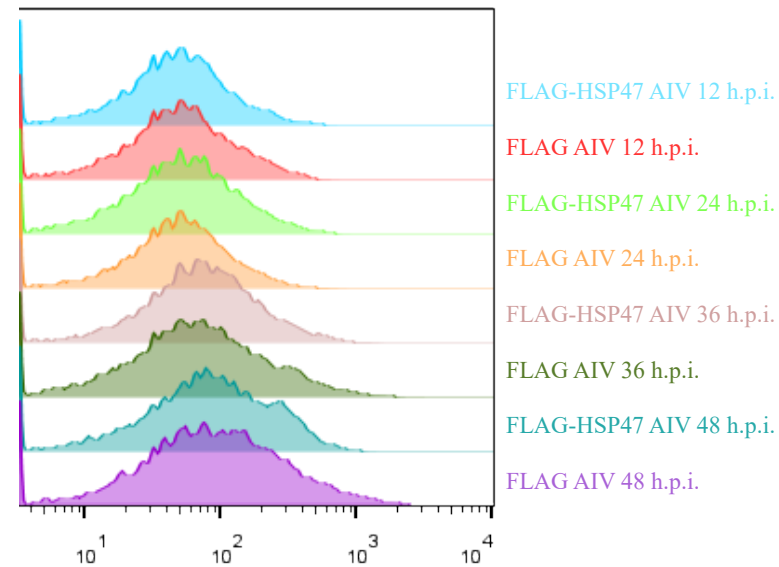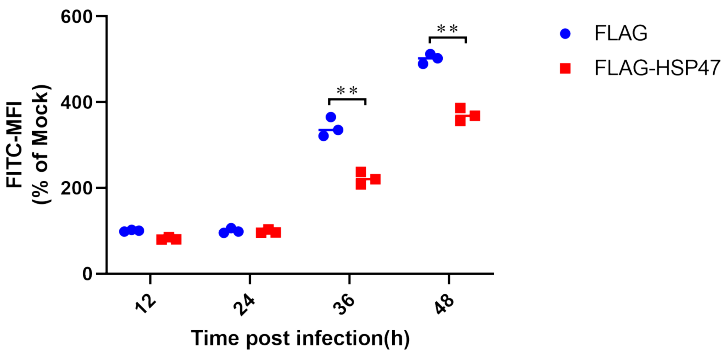

B

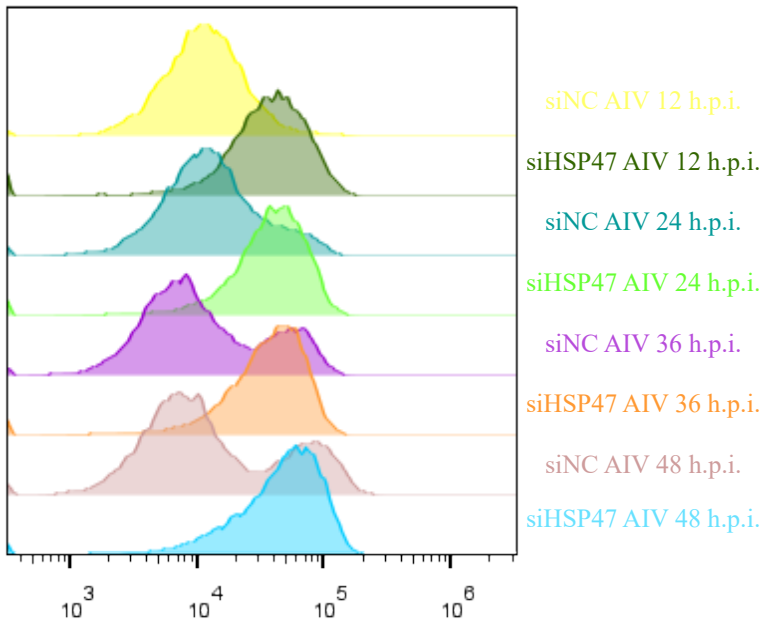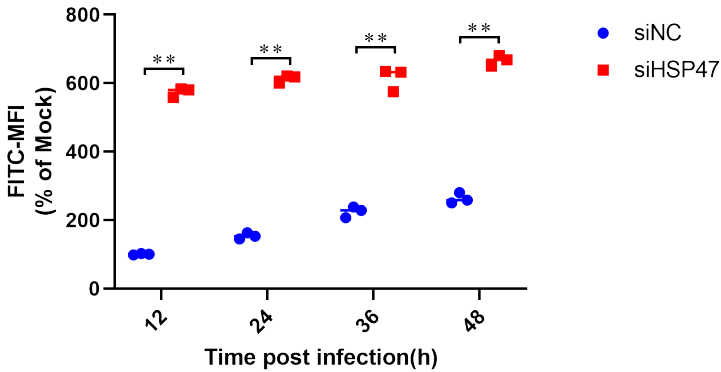

Supplement: S8 Fig — Overexpression (A) or knockdown (B) of HSP47 affects apoptosis after AIV replication in A549 cells. A549 cells were transfected with FLAG-HSP47 or FLAG-vector before infection with tested viruses. The cells were harvested at 12, 24, 36, and 48 h post-infection and stained using PI and Alexa Fluor 488 annexin V. The extent of apoptosis was determined using flow cytometry. Error bars represent SD of the mean from three independent experiments (*p<0.05, **p< 0.01) (PDF) [file ppat.1012748.s008.pdf]
