# Supplementary material for: H5 subtype avian influenza virus induces Golgi apparatus stress response via TFE3 pathway to promote virus replication
Source: PLoS Pathog. 2024 Dec 9;20(12):e1012748. doi: 10.1371/journal.ppat.1012748 (PMC11627363; doi:10.1371/journal.ppat.1012748)
Supplement: S1 Table — (DOCX) [file ppat.1012748.s009.docx]

S1 Table. Primers used in this study

| Primers | Sequence (5’-3’) | Target genes for amplification |  |
| --- | --- | --- | --- |
| qGAPDHh F | TCAAGGCTGAGAACGGGAAG | GAPDH (Human) |  |
| qGAPDHh R | CGCCCCACTTGATTTTGGAG |  |  |
| qGAPDHm F | GCGAGACCCCACTAACATCA | GAPDH (mouse) |  |
| qGAPDHm R | GGCGGAGATGATGACCCTTT |  |  |
| qGALNT5 F | GGTGTATAGCCCCCATCCCT | GALNT5 |  |
| qGALNT5 R | AGAGCTGGGAATGAGCCACA |  |  |
| qGALNT8 F | GGCAACACCCCCATCATGTA | GALNT8 |  |
| qGALNT8 R | GCATGGTTCTAAGGTGGGCT |  |  |
| qGALNT18 F | GCTTGGGATCAGGGACTCAG | GALNT18 |  |
| qGALNT18 R | GATCTGCTGACTGCTCGTGT |  |  |
| qHS6ST1 F | GCTACAACCTGTCCTTCATC | HS6ST1 |  |
| qHS6ST1 R | TGTCTTCATCCACCTCCAC |  |  |
| qB3GAT3 F | CTGACGATGACAACACCTAC | B3GAT3 |  |
| qB3GAT3 R | GCAAATCCAGCCATATCCAC |  |  |
| qGM130 F | AACCAAAGAGAGAGACGCCC | GM130 |  |
| qGM130 R | AACCGACTCCATTACCTGCC |  |  |
| qARF4 F | CACTATCTCCTCCCTCTTCTC | ARF4 |  |
| qARF4 R | TGACCACCAACATCCCATAC |  |  |
| qHSP47 F | CCGACCACCCCTTCATCTTC | HSP47 |  |
| qHSP47 R | TCGTCTCGCATCTTGTCACC |  |  |
| qTFE3 F | CCCAATATCACTGCAGGCCA | TFE3 |  |
| qTFE3 R | AGACGCCAACCACAGAGATG |  |  |
| qNP F | GGGCAGAACGTCTGACATGA | NP |  |
| qNP R | GGGTTCGTTGCCTTTTCGTC |  |  |
| qHA F | ACCTCTACGACAAGGTCCGA | HA |  |
| qHA R | CCAGTGCTAGAGAACTCGCC |  |  |
| qIL-6 F | TACCACTTCACAAGTCGGAGGC | IL-6 |  |
| qIL-6 R | CTGCAAGTGCATCATCGTTGTTC |  |  |
| qTNF-α F | GGTGCCTATGTCTCAGCCTCTT | TNF-α |  |
| qTNF-α R | GCCATAGAACTGATGAGAGGGAG |  |  |
| qIL-8 F | GGTGATATTCGAGACCATTTACTG | IL-8 |  |
| qIL-8 R | GCCAACAGTAGCCTTCACCCAT |  |  |
| qIL-1β F | TGGACCTTCCAGGATGAGGACA | IL-1β |  |
| qIL-1β R | GTTCATCTCGGAGCCTGTAGTG |  |  |
| qB4GALT1 F | TTATGCACCAGGCGGGAGAC | B4GALT1 |  |
| qB4GALT1 R | CTCCTCCCCAGCCCCAATAA |  |  |
| qMGAT2 F | TGGTGGTCCAGGTGCATAAC | MGAT2 |  |
| qMGAT2 R | GGCAGGTCTCTGGGACAATC |  |  |
| qST3GAL6 F | CTCGGCGGGTCACTCTTG | ST3GAL6 |  |
| qST3GAL6 R | GAAACCTCAGCAGAGAGGCA |  |  |
| qST3GAL1 F | CTGCGATGAGGTGGACTTGT | ST3GAL1 |  |
| qST3GAL1 R | GGCCGTCACGTTAGACTCAA |  |  |
| qRAB5 F | ACTTCTGGGAGAGTCCGCTGTT | RAB5 |  |
| qRAB5 R | GTGTCATCAAGACATACAGTTTGG |  |  |
| qRAB7 F | CAGACAAGTGGCCACAAAGC | RAB7 |  |
| qRAB7 R | AAGTGCATTCCGTGCAATCG |  |  |
| qATP6V1A F | TACCCCCACGAAACAGAGGA | ATP6V1A |  |
| qATP6V1A R | ATGATTGGCTGGCAGCTTCT |  |  |
